# Issues in pediatric haemophilia care

**DOI:** 10.1186/1824-7288-39-24

**Published:** 2013-04-20

**Authors:** Paola Giordano, Massimo Franchini, Giuseppe Lassandro, Maria Felicia Faienza, Roberto Valente, Angelo Claudio Molinari

**Affiliations:** 1 Dipartimento di Scienze Biomediche ed Oncologia Umana, Clinica Pediatrica, Università degli Studi di Bari “Aldo Moro”, Bari, Italy; 2 Dipartimento di Medicina Trasfusionale ed Ematologia, Ospedale “Carlo Poma” – Mantova, Mantova, Italy; 3 Dipartimento di Salute Mentale – ASL Bari, Bari, Italy; 4 Dipartimento di Ricerca Traslazionale e Medicina di Laboratorio – Unità Operativa Semplice Emostasi e Trombosi Ospedale Pediatrico “G. Gaslini” Genova, Genoa, Italy

## Abstract

The hemophilias are the most common X-linked inherited bleeding disorders. The challenges in children are different from that in adults and, If not properly managed, can lead to chronic disease and lifelong disabilities. Currently, inhibitors are the most severe complication and prophylaxis is emerging as the optimal preventive care strategy. Quality of life has become in the western countries the primary objective of the process of providing care, thus all the strategies (psychotherapy, physiotherapy, community life), not just the infusion of the missing factor, should be activated for the patient and family to give them the perception of being healthy.

## 

In the past three decades, haemophilia has moved from the status of a neglected and often fatal hereditary hemorrhagic disorder to that of a defined group of well-characterized molecular entities. This publication collecting the, considered by experts, most valuable bibliography is intended to be an introduction to newly care of the hemophilic children, as a small handbook for the clinical practice of pediatrician. There is little doubt at the moment that, among the most prevalent monogenic disorders (cystic fibrosis, thalassemia, muscular dystrophy), haemophilia enjoys the most efficacious and safe treatment. Indeed, after the dramatic events of widespread blood-borne virus transmission in the 1970s–1980s, there has been a strong drive towards a continuous improvement in the efficacy and safety of replacement therapy [1] and towards the cure of the disease through gene therapy [2]. Although the safety of plasma-derived factors has dramatically improved in the last 25 years, the fear related to the possible transmission by blood or its derivates of new or unknown pathogens has prompted the haemophilia treaters of western countries to treat previously untreated hemophilic babies mainly with recombinant products [3]. In pllel, with safety as a priority in mind, also the manufacturing process of recombinant factors evolved during the last few years to further minimize the risk of pathogen transmission, through the improvement of protein purification techniques, the addition of viral inactivation steps and the avoidance of human or animal proteins at any stage of their manufacturing process [4,5]. The availability for replacement therapy of safe high-quality factor concentrates was important not only for reducing the likelihood of death from haemorrhage but also for the broad implementation of prophylactic treatment regimens in order to prevent joint bleeding and resultant arthropathy, ultimately allowing patients to maintain a near normal lifestyle [6]. Quality of life has become in the western countries the primary objective of the process of providing care, which evolved from the infusion of the missing factor, the activation of all the strategies (psychotherapy, physiotherapy, community life) aimed to make the patient and family to perceive themselves as health. Prophylaxis is the gold standard for preserving joint function in babies with severe haemophilia. Primary prophylaxis is defined as regular treatment, given for at least 45 weeks of the year, initiated in the absence of documented osteochondral joint disease, determined by physical examination and/or imaging studies, and started before the second clinically evident large joint bleed and age 3 years. Secondary prophylaxis is defined as regular treatment started after two or more bleeds into ankles, knees, hips, elbows or shoulders and before the onset of joint disease documented by physical examination and imaging studies, given for at least 45 weeks of the year. Finally, tertiary prophylaxis is described as regular continuous treatment started after the onset of joint disease documented by physical examination and plain radiographs of the affected joints. The goal of prophylaxis is to maintain a FVIII concentration (FVIII:C) > 1% of normal at all times so as to avoid breakthrough bleeds. To do so usually requires the administration of FVIII 10–15 U/kg/daily or 20–40 IU/kg every second day or at least three times weekly for patients with haemophilia A and every third day or twice weekly for patients with haemophilia B. “Inter-mediate-dose protocols” are exemplified by protocols developed in The Netherlands, use lower doses administered slightly less frequently (e.g., 15–25 IU/kg, 2–3 times/week). Furthermore in these regimens doses are often adjusted according to clinical needs. However, many different protocols are followed for prophylaxis, even within the same country, and the optimal regimen remains to be defined [7]. PEDNET, the European Paediatric Network for Haemophilia Management based on the collaboration of 23 paediatricians from 16 European countries, have thus recently provided the following new definitions, precisely describing the differences between treatment schedules:

•Primary prophylaxis A is the regular continuous treatment started after the first joint bleed and before the age of 2 years while

•Primary prophylaxis B refers to regular continuous treatment started before the age of 2 years without previous joint bleeds [8].

One option for the treatment of very young children is to start prophylaxis once a week and escalate depending on bleeding and venous access. Prophylaxis is best given in the morning to cover periods of activity. Prophylactic administration of clotting factor concentrates is advisable prior to engaging in activities with higher risk of injury [7]. In the current favourable context, the most challenging complication of therapy has become the development of inhibitory alloantibodies against FVIII or FIX. These inhibitors, that develop in 25-30% of severe hemophilia A patients and in only 3-5% of those with hemophilia B, render replacement therapies ineffective [9,10]. Several issues have been reported regarding inhibitors development in children with hemophilia: host predisposition to FVIII inhibitors has been underscored by the importance of hemophilic mutation, family history, and race in predicting risk. Consequently, hemophilia genotype and immunogenetic influences have been well studied through large national and international cohorts. While null mutations in the F8 gene predispose severe hemophilia A patients to a 21-88% risk of antibody development, underlying risk diminishes to <10% in those with genotypes resulting in production of truncated nonfunctional FVIII. The inconsistency of this association when applied to the inversion mutations may now be explained by the recently implicated role of residual intracellular FVIII in modifying immunogenicity [11]. Among the many implicated treatment-related risk factors for inhibitor development in severe hemophilia A, FVIII product type, treatment intensity and FVIII prophylaxis have been the most controversial and/or the most thoroughly investigated. The retrospective CANAL study did not demonstrate an inhibitor-protective effect of pdFVIII in the treatment of children with severe hemophilia A [12]. Confirmatory data from the prospective RODIN cohort are now available [13]. Furthermore, a metaanalysis of published studies by Franchini et al. also determined that the overall prevalence of all and high titer inhibitors was not statistically different between rFVIII and pdFVIII treated [14]. The ongoing international SIPPET study is using a prospective randomized trial design to study the impact of FVIII product type on inhibitor development in minimally treated children with severe hemophilia A [15]. In a large retrospective study, intense FVIII replacement therapy administered to pediatric patients with severe hemophilia A during the first 50 FVIII exposure days was associated with an increased risk of inhibitor development, particularly when defined by five consecutive days of treatment (adjusted RR: 1.6). Furthermore, when the need for surgical prophylaxis, rather than a singular bleeding event or other indication for prophylaxis provoked the first treatment episode, the adjusted relative risk of inhibitor development was 2.6 [10]. This observation was corroborated by Maclean and associates. In an analyses of severe hemophilia A children in the UKHCDO database, 5 days of consecutive treatment was associated with adjusted odds ratios of 2.51 (P < 0.014) for all inhibitor development and 3.11 (P < 0.007) when high titer inhibitors are specifically considered [16]; lastly, in corroboration of earlier observations suggesting a protective effect of prophylaxis in smaller national cohorts [17,18], the retrospective CANAL study data further suggested an adjusted RR of 0.5 for inhibitor development in prophylaxis patients when compared to severe hemophilia A patients treated with on demand FVIII replacement [10]. Study data suggest that early prophylactic treatment and avoidance of intensive treatment periods may also protect patients from the risk of inhibitor development [19]. In Bremen and other German haemophilia centres, the implementation of a protocol of early prophylaxis, started with once weekly injections before the first joint bleed, has resulted in a very low rate of inhibitor formation [20]. Inhibitors should be screened once every 5 exposure days until 20 exposure days, every 10 exposure days between 21 and 50 exposure days, and at least two times a year until 150 exposure days. Inhibitor measurement should also be done in all patients who have been intensively treated for more than 5 days, within 4 weeks of the last infusion. Moreover, inhibitors should also be assessed prior to surgery or if recovery assays are not as expected, and when clinical response to treatment of bleeding is sub-optimal in the postoperative period [21]. The introduction of bypassing agents, such as activated prothrombin complex concentrates (APCC) (Factor Eight Inhibitor Bypassing Activity-FEIBA) and recombinant activated factor VII (rFVII, NovoSeven), has improved the management of children with inhibitors [16]. With the widespread adoption of primary prophylaxis programs and the implementation of antigen-specific immune tolerance induction (ITI) regimens requiring regular infusion of high doses of factor concentrates, the route of administration of replacement therapy has become a crucial issue [22]. Although peripheral venipuncture is the first choice, central venous access devices (CVADs) are often necessary in very young children with poor venous access. Totally implantable catheters (ports) should be preferred over external CVADs due to the lower rate of complications, especially infections and thrombosis [23]. Recently, arteriovenous fistula has been demonstrated as a reliable way to manage the venous access in haemophilic children [24]. The most likely progress in this field is the availability of FVIII and FIX molecules with longer half lives. This would be a significant step forward, considering that in countries that can afford primary prophylaxis the main obstacles to its widespread adoption are problems related to venous access. Several companies are currently developing factors with longer half-life to obviate frequent administration, and/or reduced antigenicity/immunogenicity to minimize inhibitor development [25]. The main strategies being applied to FVIII include modifications of the molecule, such as the addition of polyethylene glycol (PEG) polymers or polysialic acids, and alternative formulation with PEG-modified liposomes (PEGLip) [26]. Improved pharmacokinetics properties of coagulation factors have also been obtained through molecule modification by genetic fusion with albumin and the IgG Fc moiety [27,28]. In the last decade the ultimate goal of the investigators has been the search of the definitive cure through gene transfer of the underlying DNA defect [11,29]. The most promising clinical results in haemophilia currently are for haemophilia B, where intravenous infusion of an adeno-associated viral (AAV) vector encoding factor IX (FIX) under the control of a liver-restricted promoter has resulted in expression of FIX at plateau levels ranging from 1% to 6%, for periods of 2 years, in 6 adult males with severe haemophilia B [30]. Four of these 6 subjects, who were enrolled in the United Kingdom where twice-weekly prophylactic infusion of FIX concentrates is standard of care, have now been able to discontinue routine prophylaxis, reserving factor infusion for surgery or trauma, and the other 2 have been able to reduce the frequency of factor infusions. Those who stopped prophylaxis have largely been free of spontaneous bleeding episodes, confirming what had been predicted based on results in haemophilic dogs treated with the same approach [31] and on the natural history of mildly affected haemophilia patients. The safety of the approach to date has been excellent, with the only adverse event related to the study agent being a rise in liver enzymes (aspartate aminotransferase and alanine aminotransferase), accompanied by a decline in FIX levels, which resolved after a course of tapering steroids. Recently, interesting results of a same approach in Haemophilia A have been reported in three nonhuman primates (macaques) that showed human FVIII expression above 100% but developed neutralizing anti-FVIII antibodies that were abrogated with transient immunosuppression [32]. However AAV-mediated gene transfer to liver may have limited success in young children, as AAV vectors are predominantly non-integrating and expression is gradually lost if vector is injected into a growing animal [11]. To maintain this high level of health care and research two elements are essential. First, there is a need of more international collaborations in clinical research on haemophilia. Indeed, very few of the aforementioned cogent unsolved questions can be tackled by studies done in single, albeit large, haemophilia centers. The adequacy of the sample size is essential also for a rare disease such haemophilia and this goal can be achieved only through collaborative multicenter studies. Secondly, it is necessary to maintain a high interest and expertise in the field of haemophilia, especially among newer generations of physicians [33]. With this overall philosophy the AIEOP (Italian Paediatric Haematology Oncology Association) is promoting the care for the hemophiliac child in the associated centers. The management of hemophilia patients is a model of comprehensive care [34]. On this wave, we must be able to offer and guarantee (preferably in the same structure) services for the prevention, diagnosis, treatment and rehabilitation appropriate to the magical world of children. The hemophiliac child of the new millennium is, in western countries, a child that can have treatment options that provide to live a childhood almost similar to that of their peers, and therefore must socialize and live in community. He’s a child that can and should safety engage in physical activity. He’s a child that with his family is to be supported by a team of psychologists (developmental) experts of chronic diseases. The role of the pediatrician is crucial because it must, clutching a bond of trust with the child, be able to coordinate all care-givers to accompany his growth step by step. With the availability of prenatal diagnosis of hemophilia, the counselor must be able to provide appropriate information to the couple, and staff maternity care providers (obstetrician, neonatologist, nurses …) should be able to handle the delivery and the possible first manifestations of haemophilia. The infant is not a small man, but a puppy in evolution. A puppy who sees, in each of its psychomotor acquisition stages, change his biochemical pmeters, his consciousness of self and the others, the characteristics of his relationship with the world around him. The child with hemophilia must be adequately informed of his condition so that he can manage his path toward the adult age through school and work. Physical activity is healthy and needed for both the body and the mind. Walking, swimming, running helps to strengthen muscles and stabilize joints. Until recently, contact and high impact sports such as rugby, boxing, judo, karate has been banned to hemophilic children for the high risk of injuries that can cause bleeding; It has been reported few years ago that prophylaxis can effectively protect haemophilic children in sports that was not advised to them in the past years [35]. There is still much to investigate: gene therapy, drugs with a longer half-life (or to be given orally), immunology of inhibitors, biochemical basis of osteogenesis and joint damage [36,37]. The development of specific instruments to access the quality of life of hemophilic children [38] will allow us to better depict these points with patients’ well being in mind.

## Competing interests

The authors declare that they have no competing interests.

## Authors’ contributions

PG and GL had the idea of writing the paper. PG coordinated the group of authors. ACM wrote the final version. GL drew up the first draft and researched the bibliographic sources. MF, FFM an RV have added some comments based on their clinical experience. All authors read and approved the final manuscript.
